# Evaluation of an electronic warfarin nomogram for anticoagulation of hemodialysis patients

**DOI:** 10.1186/1471-2369-12-46

**Published:** 2011-09-26

**Authors:** Benjamin KA Thomson, Jennifer M MacRae, Lianne Barnieh, Jianguo Zhang, Elizabeth MacKay, Megan A Manning, Brenda R Hemmelgarn

**Affiliations:** 1 Department of Medicine, University of Calgary, Calgary, Alberta Canada; 2 Department of Community Health Sciences, University of Calgary, Calgary, Alberta Canada

**Keywords:** Anticoagulation, Hemodialysis, Warfarin nomogram

## Abstract

**Background:**

Warfarin nomograms to guide dosing have been shown to improve control of the international normalized ratio (INR) in the general outpatient setting. However, the effectiveness of these nomograms in hemodialysis patients is unknown. We evaluated the effectiveness of anticoagulation using an electronic warfarin nomogram administered by nurses in outpatient hemodialysis patients, compared to physician directed therapy.

**Methods:**

Hemodialysis patients at any of the six outpatient clinics in Calgary, Alberta, treated with warfarin anticoagulation were included. Two five-month time periods were compared: prior to and post implementation of the nomogram. The primary endpoint was adequacy of anticoagulation (proportion of INR measurements within range ± 0.5 units).

**Results:**

Overall, 67 patients were included in the pre- and 55 in the post-period (with 40 patients in both periods). Using generalized linear mixed models, the adequacy of INR control was similar in both periods for all range INR levels: in detail, range INR 1.5 to 2.5 (pre 93.6% (95% CI: 88.6% - 96.5%); post 95.6% (95% CI: 89.4% - 98.3%); p = 0.95); INR 2.0 to 3.0 (pre 82.2% (95% CI: 77.9% - 85.8%); post 77.4% (95% CI: 72.0% - 82.0%); p = 0.20); and, INR 2.5 to 3.5 (pre 84.3% (95% CI: 59.4% - 95.1%); post 66.8% (95% CI: 39.9% - 86.0%); p = 0.29). The mean number of INR measurements per patient decreased significantly between the pre- (30.5, 95% CI: 27.0 - 34.0) and post- (22.3, 95% CI: 18.4 - 26.1) (p = 0.003) period. There were 3 bleeding events in each of the periods.

**Conclusions:**

An electronic warfarin anticoagulation nomogram administered by nurses achieved INR control similar to that of physician directed therapy among hemodialysis patients in an outpatient setting, with a significant reduction in frequency of testing. Future controlled trials are required to confirm the efficacy of this nomogram.

## Background

Warfarin is commonly indicated as an anticoagulant for the prevention and treatment of thromboembolic diseases. However, warfarin anticoagulation therapy has been associated with increased risk of bleeding, particularly when the international normalized ratio (INR) is not maintained within the range therapeutic window [1,2]. Hemodialysis patients in particular are reported to experience a markedly increased risk of both thromboembolic and bleeding events, approximately 3 to 10 fold higher than the general population [3-6]. Warfarin anticoagulation in HD patients is also associated with an increased risk of stroke, and this risk is even higher when the INR is not routinely monitored in the HD unit [7].

Achieving anticoagulation therapy within therapeutic targets may be more challenging for hemodialysis patients due to a variety of factors including drug and dietary interactions, comorbid illness, and frequent interventions requiring reversal of anticoagulant therapy. Compared to physician guided ordering, the use of self-directed or nurse/pharmacist-guided nomograms to standardize warfarin dosing and monitoring have been shown to improve maintenance of anticoagulation in the therapeutic range, while limiting adverse events such as bleeding or thromboses, among inpatients [8-10], outpatients [11-15], and for a variety of settings [16-20].

Thus, given the effectiveness of warfarin nomograms in the general outpatient population, we evaluated whether an electronic warfarin anticoagulation nomogram administered by nurses was able to achieve similar effectiveness of anticoagulation compared to physician directed dosing, among outpatient hemodialysis patients. We also evaluated the incidence of bleeding and thrombotic events.

## Methods

### Study Population and Observation Period

All hemodialysis patients receiving stable anticoagulation therapy with warfarin (regardless of the indication), from any of the six outpatient hemodialysis clinics in Calgary, Alberta Canada were included. Patients were identified from the renal program database [21]. Two five-month time periods were considered: a pre-period prior to the implementation of the warfarin nomogram (August 1 - December 31, 2008), and a post-period, following implementation of the nomogram (March 1 - July 31, 2009). Eligible patients were included in the pre-period and/or in the post-period.

### Warfarin Nomogram

Warfarin dosing and frequency of INR monitoring for the nomogram were based on the American College of Chest Physicians (ACCP) 2008 guidelines [22] as well as guidelines from the local anticoagulation clinic. The nomogram was implemented within the renal program electronic database. A copy of the nomogram is available from the authors upon request. During the hemodialysis session, a registered nurse would enter the patients' current INR level along with their current warfarin dose. The nomogram would then instruct the nurse as to any potential adjustments to the warfarin dose and/or frequency required prior to the next INR measurement. The nomogram was designed for maintenance of anticoagulation therapy, and not for initiation of therapy. Patients initiating (or resuming) therapy had their warfarin dosing directed by a physician until their INR was therapeutic, at which point they were eligible for maintenance therapy following the warfarin nomogram. Time periods associated with the initiation of anticoagulant therapy were excluded from the study observation periods evaluating effectiveness of the nomogram.

The warfarin nomogram was introduced in the outpatient hemodialysis centers following in-servicing of the nursing staff in January 2009. A one-month transition period (February 2009) was included prior to evaluating the nomogram.

### Baseline Patient Data

Baseline demographic and laboratory data were collected from the renal program electronic database [21] which includes all patients undergoing hemodialysis in the Southern Alberta Renal Program, including Calgary. Baseline comorbidities, medications and dates of hospitalization were collected by a manual chart review of both inpatient and outpatient medical records.

### Outcome Data

The primary outcome was the proportion of INR measurements within the indicated INR range (range INR ± 0.5 units). This range was chosen to facilitate comparison of results to similar prior studies [23]. INR results for patients for both periods were abstracted from the Renal Program database. As the nomogram was developed for maintenance of anticoagulation therapy, we excluded time periods when warfarin was held or when patients were admitted to hospital, plus a 2 week period following re-initiation of warfarin or discharge from hospital. This allowed for evaluation of *maintenance *of warfarin therapy. Secondary outcomes included the mean number of INR measurements per patient, number of bleeding and thrombotic events and death, as determined by chart review. Serious bleeding events were defined as clinically overt bleeding resulting in death or hospitalization, a drop in hemoglobin greater or equal to 2 g/dL or a requirement for transfusion of 2 units of packed red blood cells. Venous thrombosis was defined as pulmonary embolus, lower or upper limb deep vein thrombosis, cerebral sinus or venous thrombosis, or visceral vein thrombosis.

### Data Analysis

Patient demographic and clinical characteristics are presented as means and standard deviations or medians and inter-quartile ranges for continuous variables, and percentages for categorical variables. Adequacy of anticoagulation before and after implementation of the warfarin nomogram was compared using generalized linear mixed models, in order to account for correlation between observations (patients may have been present in both pre- and post-periods). A similar approach was used for comparing the mean number of INR measurements for the two time periods. The analysis of the proportion of INR measurements within the range (± 0.5 units) was stratified by range INR. All analyses were performed using SAS version 9.2 (SAS Institute Inc, Cary, North Carolina) and Stata version 10.1 (StataCorp, College Station, Texas). A two-sided P value of < 0.05 was used to indicate statistical significance. The institutional review board of the University of Calgary approved the study.

## Results

### Patient Recruitment

A total of 67 patients were included in the pre-period and 55 in the post-period, with 40 patients included in both periods. Patient demographic and clinical characteristics were similar for the two periods (Table 1). The majority of subjects were on warfarin for atrial fibrillation, and over 75% of subjects had a range INR of 2.0 to 3.0. Only 2 patients in the pre- and 3 patients in the post-period had a range INR of 2.5 to 3.5.

**Table 1:** Baseline demographic and clinical characteristics

	Pre-period n = 67	Post-period n = 55
**Age, mean (SD)**	69.8 (12.8)	69.4 (14.0)
**Male (%)**	46 (68.7)	34 (61.8)
**Diabetes (%)**	34 (50.8)	33 (60.0)
**Cause of end stage renal disease (%)**		
Diabetes Mellitus	24 (35.8)	23 (41.8)
Vascular	17 (25.4)	15 (27.3)
Glomerulonephritis	9 (13.4)	3 (5.5)
Other	9 (13.4)	8 (11.9)
Unknown	8 (11.9)	9 (16.4)
**Indication for anticoagulation (%)**		
Thromboembolic disease	18 (26.9)	18 (32.7)
Cerebrovascular disease	1 (1.5)	1 (1.8)
Atrial fibrillation	31 (46.3)	23 (41.8)
Vascular access patency	4 (6.0)	2 (3.6)
Prosthetic valve	3 (4.5)	3 (5.5)
Unspecified or unknown	10 (14.9)	8 (14.6)
**Range INR (%)**		
1.5 to 2.5	14 (20.9)	9 (16.4)
2.0 to 3.0	51 (76.1)	43 (78.2)
2.5 to 3.5	2 (3.0)	3 (5.5)
**Prior bleed**	11 (16.4)	11 (20.0)
**Prior thromboembolic event**	13 (19.4)	9 (16.4)
**Known hereditary thrombotic disease**	1 (1.5)	1 (1.8)
**Use of Plavix**	0 (0.0)	0 (0.0)
**Use of NSAIDs or ASA**	1 (1.5)	1 (1.8)
**History of cancer**	10 (14.9)	5 (9.1)

INR - International normalized ratio

### Proportion of INR measurements within the range INR (± 0.5 units)

**Figure 1:**
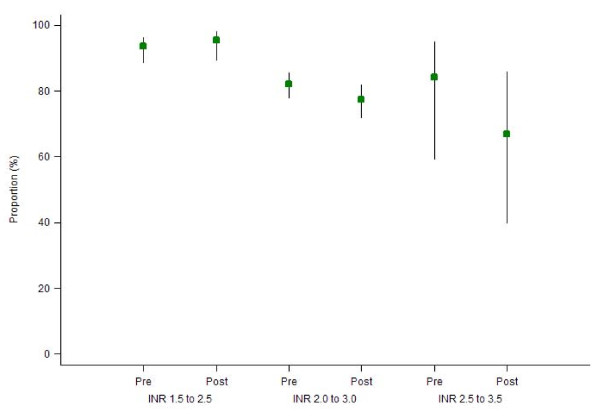
**Proportion (95% CI) of INR measurements maintained in range INR ± 0**.5 by period and range INR.

For range INR 1.5 to 2.5, the proportion of measurements within range INR in the pre- (93.6% (95% CI: 88.6% - 96.5%)) and post- period (95.6% (95% CI: 89.4% - 98.3%) (p = 0.95) were similar. Results were similar for range INR 2.0 to 3.0, with 82.2% (95% CI: 77.9% - 85.8%) within range in the pre- and 77.4% (95% CI: 72.0% - 82.0%) within range in the post-period (p = 0.20). For range INR 2.5 to 3.5, a similar proportion of INR within range in the pre- (84.3% (95% CI: 59.4% - 95.1%)) and the post- period (66.8% (95% CI: 39.9% - 86.0%)) (p = 0.29) was observed (Figure 1). Results were similar in a sensitivity analysis restricted to the 40 patients in both the pre and post-periods.

### Proportion of INR measurements precisely within the range INR

**Figure 2:**
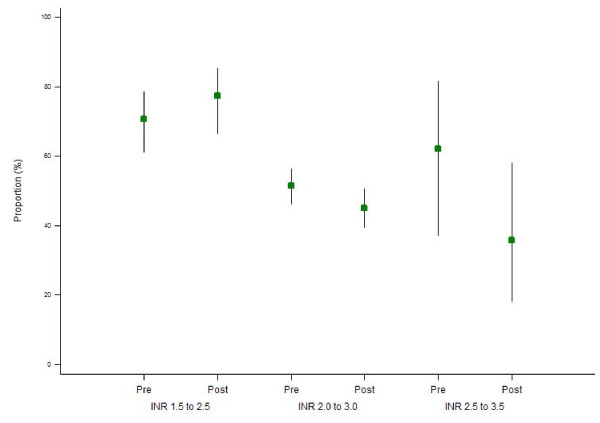
**Proportion (95% CI) of INR measurements maintained in range INR by period and range INR**.

The proportion of INR measurements that were precisely within the INR range showed similar results. For range INR 1.5 to 2.5, a similar proportion of INR measurements were within range in the pre-period (70.8% (95% CI: 61.3% - 78.8%)) and the post-period (77.4% (95% CI: 66.6% 85.5%)) (p = 0.67), while for range INR 2.0 to 3.0 the proportion within range were 51.5% (95% CI: 46.2% - 56.7%) for the pre-period and 45.1% (95% CI: 39.6% - 50.8%) for the post (p = 0.16). For range INR 2.5 to 3.5, corresponding proportions were 62.1% (95% CI: 37.3% - 81.9%) and 35.8% (95% CI: 18.3% - 58.2%) (p = 0.075) for pre- and post-period respectively (Figure 2).

### Number of INR measurements

The mean number of INR measurements per subject decreased significantly following implementation of the nomogram. During the pre-period there was a mean of 30.5 (95% confidence interval (CI) 27.0-34.0) measurements per patient compared with 22.3 (95% CI 18.4-26.1) in the post period (p = 0.0029). There were 3 bleeding events in each of the two periods. None of the bleeding events occurred at a supra-therapeutic INR, however, one of the bleeding events in the post-period resulted in death (fall with cerebral bleed) and occurred while the patient was off the nomogram, with an INR of 2.0. Overall, the number of deaths, irrespective of cause, was similar in the two time periods with 7 (10.4%) deaths occurring in the pre-period and 10 (18.2%) in the post-period. Deaths in the pre-nomogram period largely occurred out of hospital and thus were of unknown cause (6/7, 85.7%) while deaths in the post-nomogram were primarily in hospital (8/10, 80%) and for causes other than bleeding or thrombosis related. There were two thrombotic events in the pre-period (one in a patient with active malignancy) and none in the post-period.

## Discussion

To our knowledge, this is the first study evaluating the effectiveness of an electronic anticoagulation nomogram to guide warfarin therapy in the out-patient hemodialysis setting. We found that an electronic warfarin nomogram administered by nurses achieved INR control similar to that of physician directed therapy, although evidence of efficacy remain to be determined in future studies. The risk of bleeding and thrombotic events was also similar for the two methods of warfarin administration.

A recent systematic review of outpatient anticoagulation clinics reported that patients without end stage renal disease (ESRD) are within range INR range approximately 64% of the time when managed by a pharmacist or nurse guided anticoagulation nomogram [24]. However, given concerns about potential increased bleeding risk in the ESRD population, these patients are often not eligible for out-patient management of anticoagulation from an anticoagulation clinic [5,6]. Results from our study suggest that effectiveness of INR control was similar when managed by nurses using an electronic nomogram, compared to physician directed ordering. This is consistent with other non-physician directed anticoagulation management in high risk patients with complicated anticoagulation histories [23]. In hemodialysis patients, anticoagulation therapy is often confounded by multiple medications, significant comorbidity and burden of disease, nutritional deficiencies and altered pharmacokinetics due to the renal failure [25,26].

We found that the mean number of INR measurements per patient decreased in the post-period, with similar adequacy of INR control. The optimal frequency of patient monitoring and intensity of oral anticoagulation therapy is unclear. On the one hand, more frequent monitoring is not only associated with greater cost and inconvenience to the patient, but some patients with more frequent testing spend less time within a therapeutic INR, possibly due to frequent dose adjustments [27-29]. However, patients with more comorbidities and co-prescribed medications may have less stable pharmacodynamics, in which case more frequent INR measurements may be warranted [1,30,31].

Hemodialysis patients on anticoagulant therapy experience annual bleeding rates of 3.1% to 54% [6]. The annual rates of bleeding in our study were lower than previous systematic reviews published in this area, [6] and were similar for both the physician-guided or nomogram warfarin regime. Importantly the effectiveness of INR control and similar rates of bleeding in our study occurred despite a significant reduction in the frequency of INR testing. Use of the nomogram has the potential to enhance care delivery and reduce cost by tailoring frequency of repeat INR testing and allowing this aspect of care to be delivered by a nurse rather than a physician.

There is controversy related to the use of warfarin in hemodialysis patients given their increased bleeding rates and risk of hemorrhagic stroked [6,32]. The potential benefit of anticoagulation for hemodialysis patients that require anticoagulation on the basis of CHADS-2 or CHADS-VAS scoring systems remains uncertain, and a randomized control trial will likely be required before decisions regarding benefit and safety in this patient population can be made.

Our study has limitations. It was conducted in a single center, which may limit its generalizability. However, the demographic characteristics of our population are similar to the general Canadian hemodialysis population, [33] thus it would be reasonable to assume our results are also generalizable to other centers. The pre-post study design is a further limitation, though we used appropriate statistical technique to account for any potential correlation from patients present in both time periods. There is also potential for residual confounding in that we were unable to account for factors such as diet, compliance and new medications. Finally, the study size for the range INR of 2.5 to 3.5 was too small to allow firm conclusions to be drawn and any results should be interpreted with caution in this subgroup of patients.

## Conclusion

This study suggests that an electronic warfarin anticoagulation nomogram administered by nurses was able to achieve INR control similar to that of physician directed therapy among hemodialysis patients, without an increase in bleeding risk or thrombotic events. Use of the nomogram also resulted in a significant reduction in the frequency of INR testing. Evidence of the efficacy and safety of the nomogram remain to be determined in a controlled trial design.

## Competing interests

The authors declare that they have no competing interests.

## Authors' contributions

This manuscript has been seen and approved by all authors. BRH, BAK, made substantial contributions to conception, design and acquisition of data and funds. All authors made substantial contributions to interpretation of the data, all authors were involved in revising the manuscript for important intellectual content, and all authors have read and approved the final manuscript.

## Pre-publication history

The pre-publication history for this paper can be accessed here:

http://www.biomedcentral.com/1471-2369/12/46/prepub
